# Relationships between A(H1N1)pdm09 influenza infection and infections with other respiratory viruses

**DOI:** 10.1111/irv.12249

**Published:** 2014-04-04

**Authors:** Tal Meningher, Musa Hindiyeh, Liora Regev, Hilda Sherbany, Ella Mendelson, Michal Mandelboim

**Affiliations:** a Central Virology Laboratory, Ministry of Health, Chaim Sheba Medical CenterRamat-Gan, Israel; b The Mina & Everard Goodman Faculty of Life Sciences, Bar-Ilan UniversityRamat-Gan, Israel; c Department of Epidemiology and Preventive Medicine, Sackler Faculty of Medicine, School of Public Health, Tel-Aviv UniversityTel-Aviv, Israel

**Keywords:** A(H1N1)pdm09 virus, human metapneumo virus, respiratory syncytial virus, respiratory viruses, seasonal influenza viruses

## Abstract

**Background:**

A(H1N1)pdm09, a new influenza pandemic virus emerged in 2009. The A(H1N1)pdm09 infection had several unique characteristics which included rapid transmissibility and high morbidity in obese individuals, pregnant women and individuals suffering from chronic diseases.

**Objectives:**

To study the relationships between A(H1N1)pdm09 influenza infection and infections with other respiratory viruses such as respiratory syncytial virus (RSV), human metapneumo virus (hMPV), adenovirus and seasonal influenza.

**Methods:**

Samples (nasopharyngeal swabs or aspirates) collected between 2007 until 2012 from patients of various ages that were hospitalized due to respiratory virus infections were analyzed for the presence of various respiratory viruses, using qRT-PCR.

**Results:**

In 2009–2010, when the pandemic influenza A(H1N1)pdm09 first appeared, two major infection peaks were noted and individuals of various ages were infected. Following the decline of the A(H1N1)pdm09 virus infection, the percentages of patients infected with adenovirus and hMPV increased, while infection frequency with RSV B and with seasonal influenza virus decreased. Furthermore, RSV infections were delayed and very few percentages of patients were co-infected with more than one virus. Interestingly, the A(H1N1)pdm09 virus lost its dominancy when it reappeared in the winter of 2010–2011, and at this time, only the incidence of RSV infections was affected by the A(H1N1)pdm09 virus.

**Conclusions:**

The A(H1N1)pdm09 virus had distinct effects on other respiratory viruses when it first appeared versus later, when it evolved from being a pandemic to a seasonal virus.

## Introduction

Acute respiratory tract infection (ARTI) is a major cause of morbidity and mortality worldwide. Throughout the winter season, many children are hospitalized due to respiratory infections, primarily caused by influenza virus, adenovirus, respiratory syncytial virus (RSV) and human metapneumo virus (hMPV).^1^ The influenza virus is a negative-sense, single-stranded RNA virus that contains eight gene segments encoding 12 proteins.^2^ It causes a clinically distinct, systemic illness, typically characterized by abrupt-onset fever, headache, myalgia, and malaise. Similar clinical characteristics have been detected following hMPV infections.^3^,^4^ hMPV has a negative-sense single-stranded RNA genome, containing eight genes, encoding nine different proteins.^5^ RSV has a negative-sense single-stranded RNA genome, encoding 10 subgenomic mRNAs, which are translated into 11 known proteins,^6^ and is the most common cause of lower respiratory tract infections in infants up to 1 year of age.^4^ In contrast to the aforementioned viruses, adenoviruses are non-enveloped, double-stranded DNA viruses that contain 12 viral proteins.^7^ Adenovirus infections are observed throughout the year and are considered one of the most common causes of upper respiratory tract infections.^4^

Influenza infections can be life-threatening, mainly among the elderly, yet present a risk for the entire human population in the wake of influenza pandemics. Four major pandemic outbreaks have occurred in the last 100 years: the Spanish Flu pandemic (1918–1920, H1N1), the Asian Flu (1957–1958, H2N2), the Hong Kong Flu (1968–1969, H3N2), and the A(H1N1)pdm09 Flu (2009, H1N1). The A(H1N1)pdm09 virus was first detected in Mexico and subsequently spread to over 214 countries.^8^ In Israel, the virus circulated in two phases: from April 2009 until March 2010 and from October 2010 until March 2011. During 2012, only a handful of A(H1N1)pdm09 infections were reported.

The pandemic caused by the A(H1N1)pdm09 strain provided us with a unique opportunity to study the relationship between A(H1N1)pdm09 infection and infections caused by other respiratory viruses. While this relationship has been investigated by others,^9^–^11^ these studies focused on the impact of A(H1N1)pdm09 on other viruses during 2009–2010, when the virus first appeared. Here, we assessed the effects that the A(H1N1)pdm09 had on other respiratory viruses when it first appeared and later, when it became a seasonal influenza virus.

## Materials and methods

### Ethics statement

The institutional review board (IRB) of the Chaim Sheba Medical Center approved this study (Helsinki Number 9155-11-SMC). Samples (nasopharyngeal swabs or aspirates; no oropharyngeal specimens were used) were obtained from the Chaim Sheba Medical Center and from other Israeli hospitals. All diagnostic tests were performed in the Chaim Sheba Medical Center. No extra samples were specially obtained for this research. Informed consent (either written or verbal) was not required. The institutional review board waived the need for written informed consent from the participants.

### Patients and samples

The samples were all taken from patients who presented influenza-like symptoms; all samples were sent for routine clinical testing to assay for the presence of various respiratory viruses. Table1 summarizes the distribution of patients positive for the various viruses from 2007 to 2012. Tables2 and 3 summarizes the ages of the patients infected with various respiratory viruses and with influenza virus, respectively.

**Table 1:** Distribution of positive laboratory cases by virus and year

Virus	2007–2008 Pos./Total (%)	2008–2009 Pos./Total (%)	2009–2010 Pos./Total (%)	2010–2011 Pos./Total (%)	2011–2012 Pos./Total (%)
RSV	164/558 (29·4)	208/903 (23)	232/1403 (16·5)	503/2853 (17·6)*	447/2812 (15·8)*
hMPV	15/202 (7·4)	61/716 (8·5)	169/1371 (12·3)	118/2853 (4·1)	148/2812 (5·3)
Adeno	74/487 (15·2)	107/821 (13)	294/1438 (20·4)	421/2853 (14·7)	433/2812 (15·4)
Flu A**	26/495 (5·3)	79/886 (8·9)	12/16147 (0·074)	182/2853 (6·3)	142/2812 (5·1)
Flu B	45/495 (9·1)	29/886 (3·3)	16/16147 (0·099)	90/2853 (3·2)	95/2812 (3·4)
H1N1pdm	0	0	4435/16147 (27·5)	122/2853 (4·3)	21/2812 (0·7)

*P* < 0·05 for all viruses mention in this table in the various periods as compared to the reference period 2009–2010, using the chi-square test.

* The two exceptions are not significant.

** “Flu A” refers to seasonal influenza virus only.

**Table 2:** Age distribution of patients infected with various respiratory viruses

Virus	Age (years)	Before pdm 2007–2009 No. (%)	During pdm 2009–2010 No. (%)	Following pdm 2010–2012 No. (%)
hMPV	<1	5 (6·57)	46 (27·71)	15 (6)
	1–5	33 (43·42)	57 (34·33)	116 (46·4)
	6–10	2 (2·63)	8 (4·81)	12 (4·8)
	11–20	3 (3·94)	7 (4·21)	10 (4)
	21–50	16 (21·05)	22 (13·25)	20 (8)
	>50	17 (22·36)	26 (15·66)	77 (30·8)
	Total No. (%)	76 (100)	166 (100)	250 (100)
Adenovirus	<1	39 (18·57)	29 (22·48)	114 (15·38)
	1–5	109 (51·9)	77 (59·68)	506 (68·28)
	6–10	8 (3·8)	10 (7·75)	25 (3·37)
	11–20	21 (10)	6 (4·65)	32 (4·31)
	21–50	16 (7·6)	3 (2·32)	38 (5·12)
	>50	17 (8·09)	4 (3·1)	26 (3·5)
	Total No. (%)	210 (100)	129 (100)	741 (100)
RSV A	<1	54 (52·94)	131 (45·8)	230 (54)
	1–5	25 (24·5)	60 (20·97)	60 (14·08)
	6–10	1 (0·98)	16 (5·59)	14 (3·28)
	11–20	2 (1·96)	9 (3·14)	13 (3·05)
	21–50	10 (9·8)	23 (8·04)	30 (7·04)
	>50	10 (9·8)	47 (16·43)	79 (18·54)
	Total No. (%)	102 (100)	286 (100)	426 (100)
RSV B	<1	23 (48·93)	40 (50)	174 (58·78)
	1–5	12 (25·53)	27 (33·75)	42 (14·19)
	6–10	1 (2·12)	3 (3·75)	5 (1·69)
	11–20	1 (2·12)	2 (2·5)	8 (2·7)
	21–50	5 (10·63)	2 (2·5)	16 (5·4)
	>50	5 (10·63)	6 (7·5)	51 (17·22)
	Total No. (%)	47 (100)	80 (100)	296 (100)

**Table 3:** Age distribution of patients infected with influenza virus

Age (years)	Flu A No. (%)	Flu B No. (%)	H1N1pdm 2009–2010 No. (%)	H1N1pdm 2010–2012 No. (%)
0–10	159 (50·16)	80 (45·98)	692 (17·13)	63 (44·37)
11–20	23 (7·26)	9 (5·17)	591 (14·63)	11 (7·75)
21–30	17 (5·36)	7 (4·02)	764 (18·92)	14 (9·86)
31–40	6 (1·89)	15 (8·62)	524 (12·97)	8 (5·63)
41–50	5 (1·58)	9 (5·17)	489 (12·10)	6 (4·23)
51–60	10 (3·15)	19 (10·92)	435 (10·77)	16 (11·27)
61–70	34 (10·73)	6 (3·45)	350 (8·66)	8 (5·63)
71–100	63 (19·87)	29 (16·67)	193 (4·77)	16 (11·27)
Total No. (%)	317 (100)	174 (100)	4038 (100)	142 (100)

### Extraction of viral nucleic acids

Viral genomic RNA was extracted from patient samples using either the High Pure viral RNA extraction kit (Roche Diagnostics GmbH, Mannheim, Germany) or NucliSENS® easyMAG® (BioMerieux, France).

### Real-time transcription-PCR (RT-PCR) assay

Infection by the RNA respiratory viruses tested for here (influenza, RSV, hMPV, and A(H1N1)pdm09) was determined by *Taq*Man, using a panel of real-time reverse transcription-PCR (rRT-PCR) assay.^12^–^16^ For detection of the DNA virus (adenovirus), RT-PCR was performed, as previously described,^17^ using *Taq*Man Chemistry on the ABI 7500 instrument. For the RNA rRT-PCR assays, the Ambion Ag-Path master mix (Life Technologies, USA) was used, while ABgene Absolute Blue (Thermo, UK) was used for the DNA assays. The tests were performed using multiplex reactions, which involves simultaneous amplification of more than one target sequence in a single reaction. In 2007–2009, the multiplex reactions contained primers for influenza A (Flu A) and influenza B (Flu B). In 2009–2011, the multiplex reactions contained primers for Flu A, Flu B, and H1N1pdm09 and in 2011–2012, two different multiplex reactions were performed: one that included primers for RSV, Flu A, Flu B, and H1N1pdm09 and the other contained primers for hMPV and adenovirus.

### Statistical analysis

The chi-square test was applied to test the dependency/relationship between two variables (Figure 6 and Table1). All the winter season samples were compared with the winter season reference period 2009–2010, the season in which the A(H1N1)pdm09 virus had first appeared. As adenovirus infections are continuously observed throughout the year, we analyzed the average percentage of adenovirus infections from week 43 until week 19 in each year and compared it to the infections recorded during 2009–2010, the year in which the A(H1N1)pdm09 first appeared. The non-parametric Wilcoxon–Mann–Whitney Rank sum test for independent samples was used to assess the significance of these measurements. The data was analyzed by an expert statistician, using the SAS ® version 9·1 (SAS Institute, Cary North Carolina).

## Results

### A(H1N1)pdm09 affects the infection rate of other respiratory viruses

This study began with analysis of the percentage of patients infected with respiratory viruses routinely tested in our laboratory, such as seasonal influenza, adenovirus, hMPV, and RSV, between October 2007 and April 2012. The A(H1N1)pdm09 virus appeared in Israel and around the world in 2009, and therefore from 2009, we also assayed for the presence of the A(H1N1)pdm09 virus. Other common respiratory viruses, such as rhinoviruses, parainfluenza viruses, and coronaviruses were not investigated, because they are not routinely tested in our hospital. Significant changes were noted following the appearance of the A(H1N1)pdm09 virus (Table1). A minimal number of patients were hospitalized due to influenza A and B virus infections in 2009–2010 (*P* < 0·0001, compared with the other years, chi-square test). In contrast, the percentages of patients infected with hMPV and with adenovirus were significantly increased during 2009–2010 (*P* < 0·04 and *P* < 0·01, respectively), concomitant with the appearance of the A(H1N1)pdm09 infection, and returned to their usual incidence rates in the following seasons (Table1). In addition, the percentage of patients infected with seasonal influenza viruses declined sharply in the year that A(HIN1)pdm09 first appeared (Table1). However, in subsequent years, seasonal influenza infections with Flu A and Flu B were observed at normal rates. The number of patients hospitalized due to A(H1N1)pdm09 infection gradually declined with time; in 2011–2012 only a few percent (0·7%) of patients were hospitalized due to A(H1N1)pdm09 virus-associated respiratory infections (*P* < 0·0001) (Table1).

### The A(H1N1)pdm09 effect on the time of infection with other respiratory viruses

**Figure 1:**
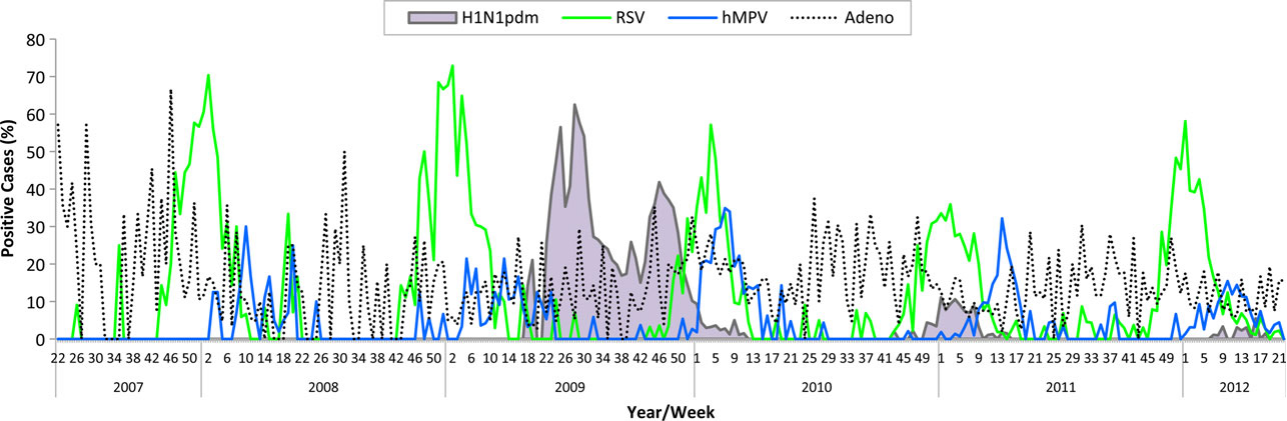
Time scale of infection with various respiratory viruses. Presentation of the weekly percent of positive cases for infection with various respiratory viruses (Adenovirus, hMPV, RSV) from 2007 to 2012. The green line summarizes infections with RSV A and RSV B. The A(H1N1)pdm09 infection is indicated as (H1N1pdm). The percentage of individuals infected with each virus was calculated each week.

To test whether the emergence of the A(H1N1)pdm09 virus also affected the time of infection with other respiratory viruses, we analyzed the weekly distribution of respiratory virus infections. Before the appearance of the A(H1N1)pdm09 virus, a hierarchy in respiratory viruses infections was observed, where the onset of RSV infections was at approximately week 43 and hMPV infections were observed between weeks 4–5 of the following year (Figure1). Interestingly, following the A(H1N1)pdm09 infection, which was first noticed in week 18 of 2009, the timing of infection with other respiratory viruses changed. In the winter of 2009–2010, RSV infections were noticed later than usual, peaking only at week 4, with the decline of the A(H1N1)pdm09 virus (Figure1). In addition, the hMPV infection peak was detected only following the decline of the RSV infection and the disappearance of the A(H1N1)pdm09 virus, in week 7. In contrast, in the winter of 2010–2011, the hMPV infection peak was observed again in week 14 (Figure1), coinciding with the time frame of its appearance in the years preceding 2009 (Figure1). Regarding adenovirus, while infections with all the respiratory viruses were not recorded until the A(H1N1)pdm09 infection declined in week 50 of 2009, the pattern of the non-seasonal adenovirus infections remained stable (Figure1). Continuous adenovirus morbidity was observed from week 49 of 2009 until week 13 of 2010, and another wave of continuous adenovirus infection was recorded from week 17 of 2010 until week 5 of 2011 (Figure1).

**Figure 2:**
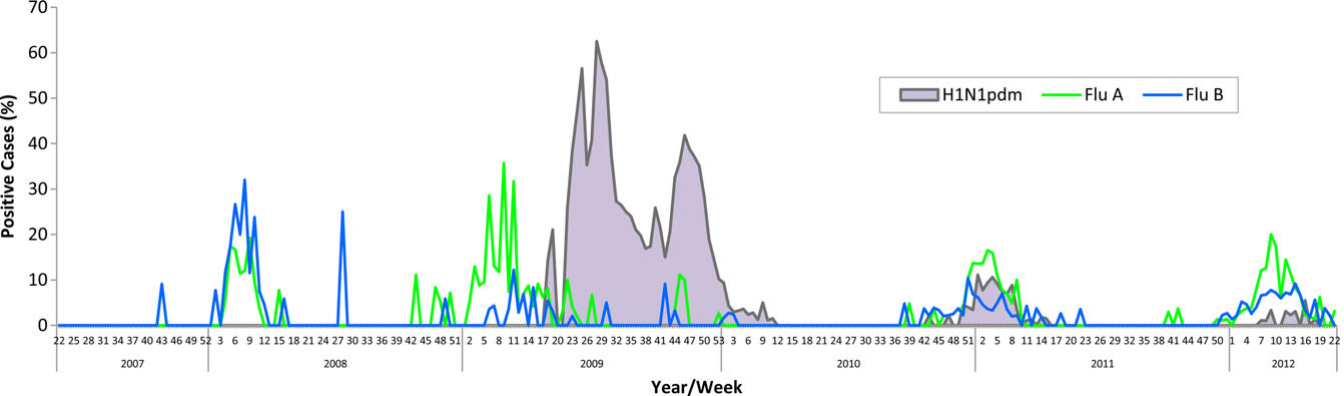
Time scale of infection with influenza viruses. Presentation of the weekly percent of positive cases of infection with various strains of influenza virus (A and B), from 2007 to 2012. The A(H1N1)pdm09 infection is indicated as (H1N1pdm). The percentage of individuals infected with each virus was calculated each week. “Flu A” includes seasonal H1N1 and H3N2 only.

In Israel, seasonal influenza infections are typically detected between January and April, peaking at the end of February, in week 9 (Figure2). Sporadic cases of influenza infections are also detected even later in the year, between weeks 42 and 50 (Figure2). In 2009, infection with seasonal influenza was detected, as usual, between weeks 1 and 9 (Figure2), immediately prior to the initial detection of the A(H1N1)pdm09 infection in week 18 of that same year (Figure2). In addition, sporadic seasonal influenza infections were also detected, despite the presence of the A(H1N1)pdm09 virus (Figure2). In contrast, in the winter of 2009–2010 (weeks 1–9 of 2010), infection with influenza (influenza A, B and A(H1N1)pdm09) was hardly detected (Figure2). In the 2010–2011, the A(H1N1)pdm09 infection was observed between weeks 1 and 8 of 2011, peaking at weeks 3–5 (Figure2). Moreover, the A(H1N1)pdm09 infection also demonstrated a biphasic pattern, with two dominating peaks between weeks 22–30 of 2009 (Figure2) and weeks 45–49 of 2009 (Figure2). This biphasic pattern was no longer observed, since 2009–2010, and the virus peak was accompanied by infection with seasonal influenza A and B viruses (Figure2 and Table1).

### Age distribution of patients infected with various respiratory viruses before, during and following the A(H1N1)pdm09 pandemic

**Figure 3:**
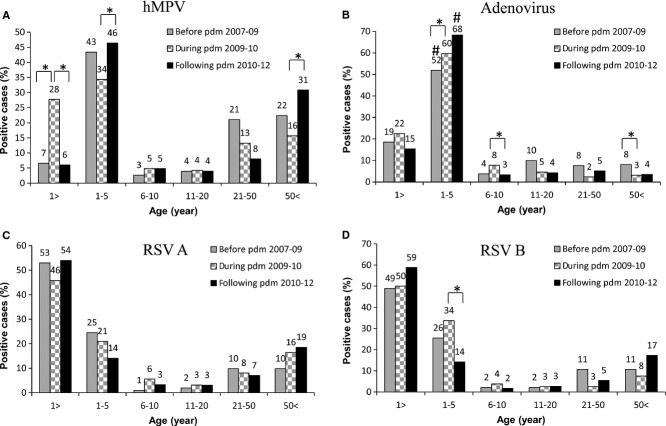
Age distribution of patients infected with various respiratory viruses. Presentation of the ages of patients hospitalized over three periods: before (2007–2009), during (2009–2010) and following (2010–2012) the A(H1N1)pdm09 pandemic. Patients were monitored for hMPV (A, 492 patients), adenovirus (B, 1080 patients), RVS A (C, 814 patients), and RSV B (D, 423 patients). For each virus, the sum of the positive cases per period was set as 100% and the percentages of the various age groups per period were calculated for each virus type. The number that appears above the columns represents the percentage of the positive cases of each virus in every age group in every period. * *P* < 0·05 using the chi-square test to compare between 2009–2010 and 2007–2009 in each age group. In addition, we compare between 2009–2010 and 2010–2012 in each age group. # In the Adenovirus graph (B) *P* < 0·05 using the chi-square test to compare between 2007–2009 and 2010–2012.

We next evaluated the age distribution of patients infected in 2007–2012. A significant elevation in the percentages of infants suffering from hMPV infection was observed during the A(H1N1)pdm09 pandemic in 2009–2010. In 2009–2010, approximately 28% of the infected children under the age of 1 year were infected with hMPV, as compared to approximately 7% or 6% before or following the pandemic, respectively (Figure3A and Table2). A significant elevation in the number of hMPV infections among patients over 50 years of age was observed and approximately 31% of the patients of this age group were infected with hMPV following the A(H1N1)pdm09 infection (Figure3A and Table2).

In contrast, the A(H1N1)pdm09 infection had a relatively small impact on the ages of patients infected with adenoviruses and with RSV A (Figure3B, C and Table2). However, a significant decline in the percentages of 1- to 5-year-old patients infected with RSV B was observed following the A(H1N1)pdm09 infection in 2010–2012, where only 14% of the children samples were infected with RSV B, as compared to 34% in 2009–2010 (Figure3D and Table2).

**Figure 4:**
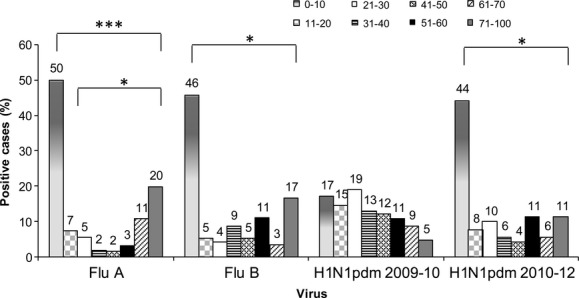
Age distribution of patients infected with the influenza virus. Presentation of the ages of patients hospitalized due to influenza infections between 2007 and 2012. Patients were monitored for influenza A (Flu A) (*n* = 317), influenza B (Flu B) (*n* = 174) and A(H1N1)pdm09 (*n* = 4180). For A(H1N1)pdm09 (H1N1pdm), two periods are shown: 2009–2010 (*n* = 4038 patients) and 2010–2012 (*n* = 142 patients). For each virus type, the sum of the positive cases was set as 100% and the percentage of the various age groups was calculated for each virus type. The number that appears above the columns represents the percentage of the positive cases of each virus in every age group. * *P* < 0·05 using chi-square test. *** *P* < 0·0008 using chi-square test.

Next, we investigated the age distribution of patients infected with A(H1N1)pdm09 and with seasonal influenza viruses between 2007 and 2012. Infections with seasonal influenza A viruses were primarily detected among patients ages 0–10 and 71–100, (around 50% and 20%, respectively, Figure4 and Table3). In contrast, influenza B virus infection was detected mostly in 0- to 10-year-old patients (46%, Figure4 and Table3). In 2009–2010, patients up to the age of 50 were equally infected with the A(H1N1)pdm09 virus and the elderly population was less infected (Figure4 and Table3). In contrast, in 2010–2012, around 44% of the A(H1N1)pdm09-infected patients were children under the age of 10 (Figure4 and Table3), an infection trend similar to that observed for seasonal influenza B viruses (Figure4 and Table3).

### Reduced percentages of co-infections following the emergence of the A(H1N1)pdm09 virus

**Figure 5:**
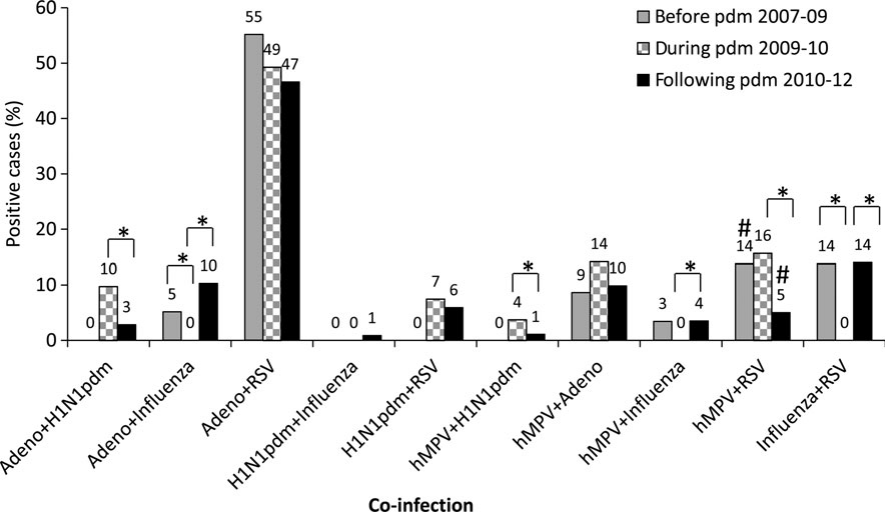
Distribution of co-infections. Presentation of the percentages of co-infection over three periods: before (2007–2009), during (2009–2010) and following (2010–2012) the A(H1N1)pdm09 pandemic. In each period, the sum of the co-infection cases was set as 100% and the percentage of the various co-infection pairs per period was calculated. The co-infection analysis was performed on samples that were tested for the presence of all viruses. */ # *P* < 0·05 using chi-square test.

We next analyzed the percentage of patients infected with several viruses, prior, during and following the A(H1N1)pdm09 infection (Figure5). Only a small percentage of patients were infected with more than two viruses (data not shown). Around 50% of the co-infections were of adenovirus together with RSV; no significant differences in the percentage of co-infections with these viruses were observed prior, during or following the A(H1N1)pdm09 pandemic (Figure5). Because seasonal influenza infections were hardly detected during the A(H1N1)pdm09 pandemic in 2009–2010 (Figure2 and Table1), the percentages of patients co-infected with seasonal influenza and with additional viruses were significantly reduced in 2009–2010 (Figure5). Overall, the percentage of patients co-infected with A(H1N1)pdm09 virus and additional respiratory virus was reduced in the years 2010–2012 (Figure5), because A(H1N1)pdm09 infection rates were lower during this period (Figures1 and 2 and Table1). Interestingly, the percentage of patients hospitalized due to co-infection with hMPV and RSV was reduced in the years following the A(H1N1)pdm09 pandemic (Figure5), likely to be due to changes observed in the timing of RSV and hMPV infection following the appearance of A(H1N1)pdm09 (Figure1).

### Clinical characteristics of A(H1N1)pdm09 infections in 2009–2010 and in 2010–12

**Figure 6:**
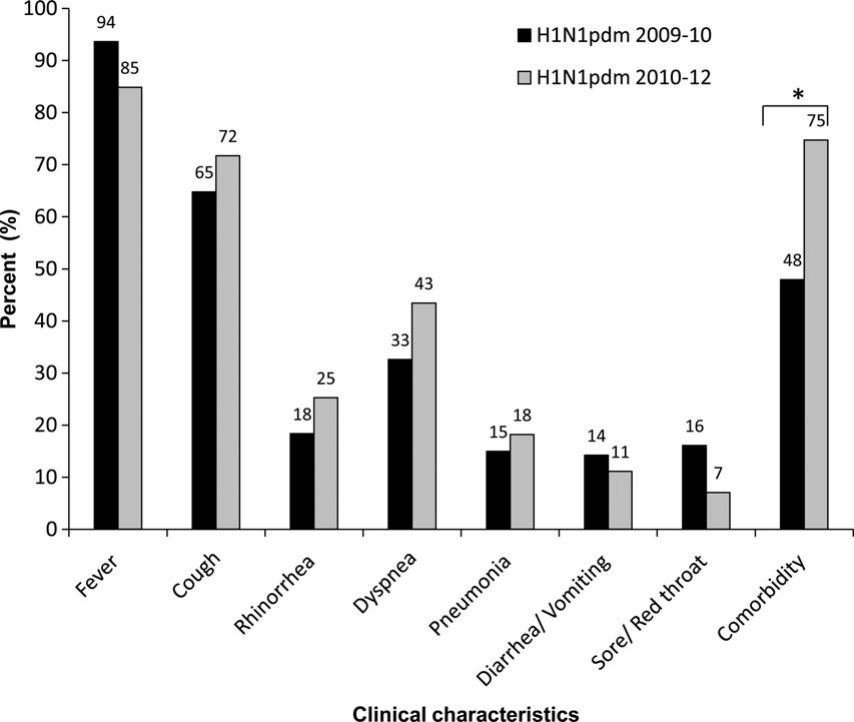
Clinical characteristics of patients infected with A(H1N1)pdm09. A summary of the clinical symptoms of A(H1N1)pdm09 (H1N1pdm)-positive patients hospitalized at Chaim Sheba Medical Center in 2009 and in 2010–2012. The number that appears above the columns represents the percentage of patients expressing the indicated clinical characteristic. * *P* < 0·05 using the chi-square test.

A(H1N1)pdm09 infection-associated symptoms among hospitalized patients were similar in both infection phases, and included fever (94% and 85% of patients hospitalized during 2009–2010 and 2010–2012, respectively), cough (65% and 72%, during 2009–2010 and 2010–2012, respectively), dyspnea (33% and 43%, during 2009–2010 and 2010–2012, respectively) and rhinorrhea (18% and 25%, during 2009–2010 and 2010–2012, respectively) (Figure6). In addition, A(H1N1)pdm09-infected patients suffered from diarrhea/vomiting (14% and 11%, during 2009–2010 and 2010–2012 respectively, Figure6), symptoms which are typically observed with seasonal influenza. Moreover, 15% and 18% of the patients in 2009–2010 and 2010–2012, respectively, had pneumonia (Figure6) and only 16% and 7% of the patients in 2009–2010 and in 2010–2012, respectively, had sore/red throats (Figure6), which is typically detected in seasonal influenza infection.^18^,^19^ In contrast, significantly more patients (75%) infected with the A(H1N1)pdm09 virus in 2010–2012 had additional background diseases (Figure6), while only 48% of the patients infected in 2009–2010 suffered from comorbidities (Figure6).

## Discussion

The presented analysis provides several important, time-dependent findings that are associated with the pandemic A(H1N1)pdm09 virus. The A(H1N1)pdm09 infection, which was first noted in Israel in 2009, circulated in the country in 2009 and 2010–2012. In 2009, a biphasic pattern of infection was observed, with peaks of infection during weeks 22–30 and weeks 45–49. In contrast, in 2010–2012, only one peak of infection was detected, during weeks 3–5. Biphasic infection patterns observed for the A(H1N1)pdm09 infection in 2009 were reported by others 20,21 and were also observed during the 1918 and 1968 pandemics,^20^ and may correlate with the school year and weather patterns. Indeed, the first 2009 peak was observed in Israel during warm weather, at the end of the school year, while the second peak was observed at colder temperatures at the end of the summer vacation.

The A(H1N1)pdm09 infection patterns differed between 2009–2010 and 2010–2012, with more patients being affected in 2009–2010, and with a greater impact on co-infection with adenoviruses, hMPV and RSV during its first phase of appearance. These disparities may have evolved from immune protection, which became more accessible between the two infection phases. In 2009, the A(H1N1)pdm09 virus was new to the population, and therefore, individuals of all age groups were infected; however, in agreement with other reports, the elderly population was less infected with the virus.^22^ In contrast, by 2010–2012, around 50% of the population had already developed immunity against A(H1N1)pdm09.^23^ During this round of infection, 44% of the patients were children under the age of 10, 58% of which were infants under the age of 1 year, born after the 2009 A(H1N1)pdm09 infection had already declined. Similar differences in the age groups affected by A(H1N1)pdm09 have been described before.^24^ We therefore suggest that the changes observed in seasonality and age distribution of the A(H1N1)pdm09 infection reflects its evolution from a pandemic to a seasonal virus. Indeed, 75% of the hospitalized patients infected with A(H1N1)pdm09 in 2010–2012 suffered from comorbidities, whereas in 2009–2010, only 48% of the hospitalized patients had additional diseases. Furthermore, in 2009–2010, the A(H1N1)pdm09 virus was the main influenza virus strain circulating in the country, replacing the previous seasonal influenza A, H1N1.

An additional interesting observation is that the A(H1N1)pdm09 virus was dominant over other influenza viruses, manifested by an influenza virus-free lag period in the winter season of the 2010–2011. While the reasons accounting for this lag period are unclear, it can be explained by a potential general anti-influenza virus immunity in the population, triggered by the 2009–2010 A(H1N1)pdm09 infection, that prevented subsequent infections in 2010–2011. In the beginning of 2011, the seasonal influenza and A(H1N1)pdm09 infections were noted at the same time, an observation also seen in other countries.^25^,^26^ In addition, like in other countries, the only seasonal influenza A observed as the appearance of the A(H1N1)pdm09 was seasonal H3N2, while the seasonal H1N1 has disappeared altogether since then.^27^ The dominance of the A(H1N1)pdm09 infection did not affect co-infection with RSV and adenovirus, which is the most common co-infection.^28^,^29^

We also observed that infection with the A(H1N1)pdm09 virus influenced the infection pattern of other respiratory viruses, especially in 2009. The time of the infection with other respiratory viruses changed and hMPV and adenovirus infections were more frequent only after the A(H1N1)pdm09 infection had declined. Increased hMPV infection rates were noted in infants <1 year of age. Furthermore, in agreement with other studies,^30^,^31^ following the A(H1N1)pdm09 infection, RSV infections were detected later than usual. We also observed, as reported in Hong Kong,^32^ that children between the ages of 1–5 years were less frequently infected with RSV B following the decline of the A(H1N1)pdm09 infection. The reasons for this decline are not completely understood, but can plausibly be due to the generation of a cross-reactive specific and non-specific anti-RSV B immunity following the A(H1N1)pdm09 infection. Indeed, it has been well established that influenza virus-infected cells produce interferon and other cytokines.^32^,^33^ Other studies have suggested that the impact of the A(H1N1)pdm09 infection on RSV immunity may be the result of increased hygienic measures implemented following the pandemic.^30^,^31^ Regardless of the reasons accounting for the reduced RSV infection following the A(H1N1)pdm09 pandemic, these results suggest that pandemic influenza infections may provide some minor benefits by reducing infection rates of other viruses.

In conclusion, we demonstrate here that the A(H1N1)pdm09 virus significantly affected infections with other respiratory viruses, when it first appeared in 2009. We further showed that in subsequent years, the A(H1N1)pdm09 virus lost its dominancy, suggest to be due to its evolvement from a pandemic virus into a seasonal influenza virus.
